# Supplementary material for: Tracking district-level performance in the context of achieving zero indigenous case status by 2027
Source: PLOS Glob Public Health. 2023 Jan 6;3(1):e0001292. doi: 10.1371/journal.pgph.0001292 (PMC10021673; doi:10.1371/journal.pgph.0001292)
Supplement: Attachment — Submitted filename: Response to Reviewers.docx [file pgph.0001292.s001.docx]

#################################################################################

**Reviewer-1**

#################################################################################

Please use the space provided to explain your answers to the questions above. You may also include additional comments for the author, including concerns about dual publication, research ethics, or publication ethics. (Please upload your review as an attachment if it exceeds 20,000 characters)

Reviewer #1: Apart from the specific comments mentioned in the attached comments file, I have the following comments to the authors:

**Response:** Specific comments mentioned in attached pdf file have been answered in word documents that can be seen in track change mode.

**1. Do the authors think that the decline in malaria cases is a linear event? More so, when the remaining cases are very few? As per my observations, as the number of cases decease, the decline tends to be non-linear. And therefore, appropriate models should have been used for different districts based on the declining trends.**

**Response:** We are very thankful to the reviewer for raising this concern and we fully agree with the reviewer that the decline tends to be non-linear. We have attempted different methods of analyses on these data and we observed that the decline in malaria cases is non-linear. The reasons could be many including heterogeneity in Indian districts in terms of endemicity, transmission intensity, differential control interventions, different climatic zones, migration rate and others. Moreover, the rate of change in the malaria cases is also dynamic over time as different control interventions have been incorporated in malaria control program at different time points and their intensity over the period varies. Therefore, the use of standard statistical methods such as Generalised Liner Model (GLM), Time series regression (such as AR, MA, ARIMA, SARIMA etc.), Smoothing techniques (such as single, double or triple smoothing), machine learning method (such as random forest, xgboost etc.) has not been used as they require data on large number of variables (such climate data and intervention data, monthly malaria cases) at a granular level which is not available for all the Indian districts. On the other hand, the process of forecasting using these methods is highly complex which is not intuitive. Therefore, in this study, instead of forecasting we had projected the number of years to reach zero case status by studying the recent progress in malaria caseload reduction via extrapolating. We have used relatively simple and robust methods which are highly intuitive to all and require lesser data and yet are powerful in identifying those districts which are not on track to achieve zero cases status by 2027. We have flagged the districts whose recent progress is not encouraging as per the stated goal. This will allow stakeholders to modify their control strategies to achieve the goal of zero cases status by 2027.

Regarding the issue of districts where malaria cases in last four years are few, will require deeper analysis of multiple predictors specific to that district. Such information is readily available and deviates from the central theme of the paper. The present methods will not be applicable to the district with only few cases of malaria are there as the rate of change is steep and may not be reliable. Therefore, in this study we have excluded those districts where average cases in last four years is less than 50.

**2. The toughest part on the road to zero cases is the last few cases and the authors have excluded such districts with less than 50 cases. How would you defend that?**

**Response:** Yes, we agree with reviewer that last mile in the road to elimination is the toughest part and needs its due attention. As discussed above, districts where are cases are few (or below 50) require specialize modelling which require district-specific data that are not available presently. Our focus is on high malaria burden districts because they are more crucial in the present scenario for achieving malaria elimination by 2027. It has been added in line number 1-5 on page number 5 in clean version of manuscript.

**3. Taking the last 3 years trajectory is not optimal to predict the year for achieving a zero case, specially when 2020 was badly hit by COVID. I would suggest to use a longer period of observations for making such predictions**

**Response:** Our primary objective of the study was not to predict the year of achieving zero case status but was to project year of elimination based on recent progress in case load reduction. In general, recent progress can be assessed by studying three to five years’ data and here we have used four years’ data (2017 to 2020) to calculate three yearly-rate-of-reductions in caseload (caseload rate of reduction in 2018 compared to 2017, caseload rate of reduction in 2019 compared to 2018 and caseload rate of reduction in 2019 compared to 2020). As per the national program, there was little impact of COVID on malaria surveillance in India. Therefore, our results will be biased if we straight away remove 2020 data from the analysis based on an assumption disruption of malaria activities due to COVID. Equally, data before 2017 have not been included as India launched the National Framework for Malaria Elimination in 2016 and National Strategic Plan (NSP) and presented a detailed work plan at the district level in 2017. With the deployment of NSP, India shifted from control to elimination and programmatic activities pertaining to surveillance and disease control were re-oriented accordingly. Thus efforts towards malaria elimination were significantly ramped up in the country in 2017. Including data before this period greatly increases the bias in the estimations, underestimating the effect of recent interventions by a large margin, and thereby makes it difficult to pin point the most vulnerable districts that need special focus. It is for this reason that we decided to use last four years data that takes into account the increase in intervention efforts, and helps to identify districts where intervention efforts need to be increased even further. Therefore, for the purpose of our analysis, we have analysed four years’ district level data from 2017 to 2020.

4. **How did the authors assessed the validity of their predictions?**

**Response:** As discussed above, our primary objective of the study was not to predict the year of achieving zero case status but was to project year of elimination based on recent progress in case load reduction. Model validation is not applicable here as we have not developed any prediction model (we have discussed this in limitation as well). We have just assessed the past performance by calculating simple statistics on their past rate of reduction in caseloads.

5. Why the average of RoR was made and why not Y3-Y1 data taken into account?

**Response:** In our study, we have assessed the districts’ recent performance in caseload reduction with respect to the goal of zero case status by 2027. The performance of a district was evaluated by calculating the annual percentage change in the total number of malaria cases for the years 2018, 2019, and 2020 considering the previous year as a base year by using the following formula

Annual % change in malaria cases for 2018 = $\frac{Malaria cases in 2018-Malaria cases in 2017}{Malaria cases in 2017}*100$

Annual % change in malaria cases for 2019 = $\frac{Malaria cases in 2019-Malaria cases in 2018}{Malaria cases in 2018}*100$

Annual % change in malaria cases for 2020 = $\frac{Malaria cases in 2020-Malaria cases in 2019}{Malaria cases in 2019}*100$

Once the annual change is calculated, the Mean Rate of Change (Mean RoC), Median Rate of Change (Median RoC) and Maximum Rate of Change (Max. RoC) were calculated on these changes.

For example, suppose we have a district named 'A' where the annual reduction rate in 2018, 2019 and 2020 were -23.56%, -31.51% and -25.40%, respectively and total cases in 2020 were reported as 6734. The mean RoC, median RoC, and Maximum RoC can be calculated as

Mean RoC =Average (23.66, 31.51, 25.40) = 26.83

Median RoC =Median (23.66, 31.51, 25.40) = 25.40

Maximum RoC =Maximum (23.66, 31.51, 25.40) = 31.51

If we consider the best-case scenario, 31.51% will be the Rate of Reduction (RoR). Based on this rate of reduction, the projected cases in 2027 can be calculated as

Cases in 2020	:	6734

Cases in 2021	:	6734-6734$*\frac{31.51}{100}$ = 4612

Cases in 2022	:	4612-4612$*\frac{31.51}{100}$= 3159

Cases in 2023	:	3159-3159$*\frac{31.51}{100}$= 2163

Cases in 2024	:	2163-2163$*\frac{31.51}{100}$= 1482

Cases in 2025	:	1482-1482$*\frac{31.51}{100}$= 1015

Cases in 2026	:	1015-1015$*\frac{31.51}{100}$ = 695

Cases in 2027	:	695-695$*\frac{31.51}{100}$ = 476

The above calculation can be rewritten in a simplified form using the compound interest formula given below:

$$\boldsymbol{Projected Malaria Cases in 2027=Malaria cases in 2020 \times}\left( \boldsymbol{1+r} \right)^{\boldsymbol{7}}$$

Where *'r'* is the RoC in percentage (negative to indicate reduction) and 7 is the time component (from 2020 to 2027). All values were rounded off to 0 decimal places to denote whole cases. For example, Projected Malaria cases in 2027 in district A will be 6734*(1-0.3151)^7^ =476.06174~=476

As reviewer asked for if instead of working on ROC if we would have worked on caseload difference, the above discussed approached could not be followed.

6. Why wasn’t a regression model used to fit the data taking the slope of the decline into account?

**Response:** As discussed above, our primary objective of the study was not to predict the year of achieving zero case status but was to project year of elimination based on recent progress in case load reduction that can be done by following above discussed approached only. Therefore, no regression model was used and other than these for better estimation by the regression model multiple data are needed which is available in public domain.

7. The reference numbers 24 and 28 are the same. Please use caution while reporting

**Response:** Thanks for noticing it, it has been corrected now and manuscript has been thoroughly checked.

8. Authors' contributions need to be mentioned in the ICMJE format

**Response:** Author’s contribution has been modified as ICMJE format.

9. The figure quality is very poor and barely readable. Please provide high quality figures

**Response:** High Quality images have been uploaded now.

10. Figure 1: it says <50 cases under exclusion and that includes 0 case as well. Please use appropriate words. The project year of elimination should be replaced with year of achieving zero case. The year range mentioned is 2027-2030 - I suppose this should be 2028-2030? The post-2030 event should be broken down into 5-year brackets for more granularity. Why is there an arrow dropping from the Post-2030 bubble?

**Response:** We are very thankful to the reviewer. The corrections have been incorporated into the figure. The year range 2027-2030 has also been rightly corrected to 2028-2030. Also, the increasing incidence bubble has been moved up. Splitting the flowchart any further makes it extremely large, complex and indiscernible. The corrected figure will be uploaded with the revised manuscript.

11. Figure 2c: The year range 2028-2030 should reflect 2027-2030? I am confused

**Response:** As per the comments on Figure 1, since the estimation is for years after 2027, the range should be 2028-2030. This has been corrected in Figure 1, and in Figure 2 as well. Moreover, “Projected zero case status before 2027” should read as “by 2027”. The corrected figure will be uploaded with the revised manuscript.

12. Figure 3c: the legend is confusing and is incomplete. Elimination should be replaced with zero case. Please add the name of the correspond state or UT against each district mentioned.

**Response:** As suggested, elimination has been replaced with zero case status and corresponding status and UT of each district has been added in the legend.

13. Figure 4: How did you estimate the required RoR as nothing is mentioned in the methods. The required RoR cannot be depicted as a line diagram as the data is non-continuous. Please arrange the districts as per ascending/descending order of Max RoR and mention the respective states/UTs. How is it possible that a district with higher Max RoR (Vishakhapatnam) as compared to Shahjahanpur has a higher required RoR 69.6 as compared to 63.4?? Same is the case with Junagarh and West Tripura.

**Response:** Calculation of required RoC has been added to the methods section. Please note that the required rate of change depends upon two factors: present rate of change and caseload in each year.

14. Figure 5: legend is incomplete

**Response:** The Legend has been updated to include the names of the districts (States) represented in the graphs.

#################################################################################

**Reviewer-2**

#################################################################################

**Review of Yadav et al paper - ‘Tracking malaria cases in Indian districts in the context of achieving zero indigenous case status by 2027’**

**Major comments:**

The paper is interesting, and important in terms of potentially targeting resources to ensure country elimination targets are met. The results should be useful to national and state level programs when considering how to further target resources to ensure elimination targets are met – and because the methods are relatively simple and transparent, this could aid overall uptake by decision makers. However, the results and discussion as to activities that districts should pursue to achieve their elimination targets seemed generic and applicable to many countries currently facing rising malaria cases.

The paper could be strengthened in several suggested ways;

Firstly, regarding the methods in the paper.

1. **Could authors explain the rationale for using 3 years of data?**

**Response:** Our primary objective of the study was not to predict the year of achieving zero case status but was to project year of elimination based on recent progress in case load reduction. In general, recent progress can be assessed by studying three to five years’ data and here we have used four years’ data (2017 to 2020) to calculate three yearly-rate-of-reductions in caseload (caseload rate of reduction in 2018 compared to 2017, caseload rate of reduction in 2019 compared to 2018 and caseload rate of reduction in 2019 compared to 2020). As per the national program, there was little impact of COVID on malaria surveillance in India. Therefore, our results will be biased if we straight away remove 2020 data from the analysis based on an assumption disruption of malaria activities due to COVID. Equally, data before 2017 have not been included as India launched the National Framework for Malaria Elimination in 2016 and National Strategic Plan (NSP) and presented a detailed work plan at the district level in 2017. With the deployment of NSP, India shifted from control to elimination and programmatic activities pertaining to surveillance and disease control were re-oriented accordingly. Thus efforts towards malaria elimination were significantly ramped up in the country in 2017. Including data before this period greatly increases the bias in the estimations, underestimating the effect of recent interventions by a large margin, and thereby makes it difficult to pin point the most vulnerable districts that need special focus. It is for this reason that we decided to use last four years’ data that takes into account the increase in intervention efforts, and helps to identify districts where intervention efforts need to be increased even further. Therefore, for the purpose of our analysis, we have analysed four years’ district level data from 2017 to 2020. Discussed in page number 5, line 1 to 5 in clean copy of manuscript.

2. Authors could consider validating the method used to identify districts that require focus. For instance, taking case numbers from the three years prior to 2017, applying the same method and determining how results compare to reality from 2017 – 2020 would be a good exercise to allow for greater confidence in results.

**Response:** Yes, we can understand reviewer’s concern, we would like to bring to your kind notice that we have not developed any statistical model to forecast number of malaria cases at district level by 2027. In our study we have assessed districts’ recent performance in caseload reduction with respect to the goal of zero case status by 2027. The performance of a district was evaluated by calculating the annual percentage change in the total number of malaria cases for the years 2018, 2019, and 2020, considering the previous year as a base year by using the following formula

Annual % change in malaria cases for 2018 = $\frac{Malaria cases in 2018-Malaria cases in 2017}{Malaria cases in 2017}*100$

Annual % change in malaria cases for 2019 = $\frac{Malaria cases in 2019-Malaria cases in 2018}{Malaria cases in 2018}*100$

Annual % change in malaria cases for 2020 = $\frac{Malaria cases in 2020-Malaria cases in 2019}{Malaria cases in 2019}*100$

Once the annual change is calculated, the Mean Rate of Reduction (Mean RoR), Median Rate of Reduction (Median RoR) and Maximum Rate of Reduction (Max. RoR) were calculated on these changes. After this projected number of years required to reach the goal of zero indigenous cases if mean of the last three years’ cases reduction is maintained (Scenario 1) and if the median of the last three years’ cases reduction is maintained (Scenario 2) and the third scenario where what happens if they work on their maximum cases reduction in last three years. Since we have not developed any regression model, therefore, validating the result taking data prior to 2017 may not be applicable. Our approach is simple and intuitive and very useful for programme perspective for tracking progress of districts.

3. Additionally, authors could do some sensitivity analysis to identify whether increasing the number of years or weighting more recent years could improve predictions.

**Response:** As discussed in above two responses, here our objective is not to predict malaria cases but to develop algorithm to assess current progress of the districts in context of goal of zero cases by 2027, to identify districts where the current rate of change (and in association the intervention efforts) are not sufficient. Therefore, validity and sensitivity analysis is not applicable in the context of present objectives.

4. It is not clear whether the data is used is only from the public sector facilities? Assuming it is, could authors comment on the utilisation of the private sector by patients and how this impacts the findings? This would tie in with the discussion challenges which mention the private sector.

**Response:** The data used for this paper is from public sector which is collected a by the National Centre for Vector Borne Disease Control (NCVBDC), nodal agency of Government of India for control of vector borne diseases. The malaria surveillance of the country uses the network of health care services from primary to tertiary level and the data is collated from grassroots (village) to tertiary (district) level. However, the surveillance of the Indian malaria control programme is considered weak as it captures ~8% of the total case load as per World Malaria Report 2018. A substantial case burden is in private sector (both formal and informal) which does not get reflected in the national figures (please see refs below: 1 and 2). Since the number of cases reported by the national malaria programme are only considered by the government in gauging the progress towards malaria elimination and considering the ambiguity in the case load contributed by private sector, it was prudent that only reported cases were utilized for analysis in our paper.

We agree with the reviewer that large unknown case load in the private sector could influence our findings and impact the accuracy of our analysis. Since we do not even have a rough estimate of the malaria cases in private sector, the extent of its influence on our analysis would be difficult to judge.

Opacity of private sector and its contribution to malaria management despite malaria being made a notifiable disease is definitely a huge challenge and is discussed in the paper.

^1^ Sharma, S., Verma, R., Yadav, B., Kumar, A., Rahi, M., & Sharma, A. (2022). What India can learn from globally successful malaria elimination programmes. BMJ Global Health, 7(6), 8431. <https://doi.org/10.1136/bmjgh-2022-008431>

^2^ Rahi, M., & Sharma, A. (2022). Perspective Piece Active Engagement of Private Healthcare Providers Is Needed to Propel Malaria Elimination in India. American Journal of Tropical Medicine and Hygiene, 106(6), 1585–1588. https://doi.org/10.4269/ajtmh.22-0025

5. It would be good to state the proportion of cases diagnosed via RDK vs microscopy given that conventional RDTs do not have good sensitivity for vivax infections (i.e. not sub-microscopic or asymptomatic). If most cases are diagnosed using RDK or if there has been a shift with more emphasis on RDK use in the last 3 years in some districts, what are the implications of this on the results. This should also be addressed in the limitations.

**Response:** We thank the reviewer for highlighting this point. The majority of the cases are still diagnosed by microscopy in India, which is considered as the gold standard for malaria diagnosis as per the National Centre for Vector borne Disease Control (NCVBDC) ^1^. Where microscopy is results are available within 24 hours, it is the only routine method for malaria diagnosis used, whereas RDTs are generally used only for emergencies where accessibility is a problem. Even when RDT is used for testing, the guidelines state that the confirmation of positive cases has to be done by microscopy^1^. Based on these guidelines, we can assume that almost all the cases reported by NCVBDC have been confirmed by microscopy. India procures about 14 million RDTs annually though data on cases detected from RDT are not available.

In our analysis we have simply addressed the trends in reduction of cases without investigation the operational details in context of types of diagnostics or any other intervention measures like distribution of LLIN, IRS etc.. These clearly influence the epidemiology of malaria in India but are not the central theme of our work here. Indeed, the use RDTs or other measures of surveillance or control are as per the national policy (based on stratification of districts as per malaria burden) could possibly influence the malaria incidence.

^1^ NCVBDC. (2014). Guidelines for Bivalent RDT. https://nvbdcp.gov.in/WriteReadData/l892s/guidelines-for-bivalent-rdt.pdf [Accessed 7/10/2022]

6. Recognising that this could be challenging – therefore just a suggestion for consideration, if data is available from the state programs on resource allocation over the last three years to the districts that are predicted to fail – that could be a proxy for intensity of malaria activities in those areas and enable the authors to highlight where resources need to be re-allocated to improve the changes of the NVBDCP achieving their stated targets.

**Response:** We thank the reviewer for this insightful suggestion. Indeed, the resources spent or allocated to different programme activities could be good indicator of the focus given by state Govt. to certain aspects of malaria control programme but unfortunately we do not have access to that data at any level, national, state or district. Hence this paper will be unable to address this suggestion as the data are not available at any scale.

Epidemiology of malaria in India

7. It would be helpful to provide more detail on malaria epidemiology in India in 2020 especially related to the point about RDKs, but also because we know historically the lag between falciparum and vivax elimination could be quite long. Additional epi information that would be useful for background could include for example, the proportion of Pf to Pv., potentially the distribution of G6PD deficiency, the status of the districts in which elimination has been achieved or reported <50 cases between 2017 – 2020. e.g. are they receptive for malaria and for how long have they been in POR phase?

**Response:** Total of ~23.88 million RDTs / 0.12 million cases were diagnosed using RDT and 73.3 million microscopic blood smear examined and 0.065 million cases were detected in 2020 (Annexure 5H, World Malaria Report-2021). Overall, the distribution of plasmodium species P. falciparum and P.vivax has been approximately in equal distribution in earlier years and in 2020 the proportion of Plasmodium vivax was 36%. Prevalence of G 6 PD deficiency (particularly in tribal populations) varies from 2.3 to 27.0 per cent with an overall prevalence of 7.7 per cent in different tribal groups. It is appreciated that vivax malaria would be a bigger roadblock in our journey towards malaria elimination due to several reasons such as liver stage hypnozoites which can trigger fresh infections, failure of compliance to 14 days radical cure with primaquine (PQ), G6PD deficiency contraindicating treatment with PQ, questionable robustness of RDTs in diagnosis of vivax malaria amongst others. Receptivity has been defined by WHO as the degree to which an ecosystem of an area permits transmission of Plasmodium spp. from a human through a vector mosquito to another human. This includes vectorial capacity, susceptibility of the community to infection, and the ongoing malaria interventions. However, there is no single or standard measurement of receptivity (Yukich May 2022 Malaria Journal). The above factors have not been assessed for the districts with < 50 cases or any other category of districts. The central theme of this paper is to focus attention on to Indian districts where appreciable reduction in malaria caseload is presently not being achieved as this will derail plans for elimination of malaria but the stated deadline.

8. Objective: Suggest that the key objective is stated in the abstract and introduction ie. identifying the most-off track districts (as opposed to tracking overall change in all districts which explains the exclusion of POR and near-elimination districts). It would be helpful to have a table in the results section that provides a summary information on the number of districts set to achieve various targets under the mean, median and maximum cases.

Response: As suggested, the key objectives have been mentioned clearly in the abstract (Page 2: Line 5-6) and in the introduction section (Page 4: Lines 18-20). The table providing the summary of the districts set to achieve the analysed targets have also been added (Table 1).

9. Discussion section: Are the challenges (private sector, inaccessible tribal regions, asymptomatic reservoirs and sub-microscopic cases, patient adherence and LLIN coverage) outlined in the discussion section related specifically to the districts and states in which the changes of elimination are lowest? If not, could there be a more in-depth analysis of the challenges specific to those districts identified as least likely to achieve elimination? Eg is there information on the LLIN coverage in the districts least likely to achieve elimination? Is vivax more prevalent in those districts? Are they border or areas with accessibility issues?

Response: While the challenges outlined in the discussion do have direct relevance for the vulnerable districts identified in our study, they are also evident in other districts with different burden of malaria, and are not specific to these districts only. The vulnerable districts identified in our study have significantly different burden of malaria, geography and resources. Therefore, the causes of sustained transmission and inadequate impact of intervention differ significantly among these districts. Identifying the underlying causes would require detailed local analyses but the relevant data are not available in the public domain. Data on LLIN coverage needs to be procured from the local malaria control wings. To identify issues with accessibility, it would be necessary to identify functional Sub Health Centres (SHCs) and Primary Health Centres (PHCs) in the vicinity as well as the availability of adequate personnel and resources in these government health centres. Such local level analysis is beyond the scope of the current study as the data are simply not available in the public domain. However, as the reviewers have rightly suggested, future research can be focused on identifying the underlying causes of missing the zero case status target, and we thank the reviewers for their valuable suggestion regarding this.

10. Related to the point above on the challenges – it seems that, in tandem, routinely identifying symptomatic infections (for vivax) in addition to asymptomatic cases (assuming falciparum) is also key.

**Response:** We agree with the above suggestion, and studies are currently being undertaken at our institute to differentiate the asymptomatic burden in India as compared to the symptomatic infections which could reveal important insights on this.

11. Overall, the solutions for the challenges identified are complex to set into policy and implement between now and 2027. My question is are the presented solutions really feasible? Taking the suggestion of implementing TQ as a solution for patient adherence as an example, that would require implementing G6PD testing. Drug registration can take 3 years or longer, policy change a further 3 years (Varunika et al, 2021) and we know from other countries in the region that preparation and then targeted scale-up of G6PD testing alone takes about 1.5 years. If all started in 2023, that would bring us to 2028 to implement solutions that are potentially meant to assist districts move to certification a year earlier. This likely applies to many of the solutions presented. My point is that either the solutions are not feasible without strong will to implement as soon as possible, or the elimination targets are not feasible. A comment for the authors consideration as to whether to discuss this or not.

**Response:** The reviewer has pointed out to a very pertinent issue of delay in translation of research into policy. However, we are of the opinion that the solutions offered in the paper are feasible if the policy makers recognise the challenges and accept the possible solutions and make urgent efforts to explore the deployment of the same. The success of TQ and use of TQ in other countries should pave the way for its registration and use in programmatic conditions under research mode in India intially. It has been shown to be safe and efficacious in Phase 2a/b trials in India ^1^. TQ has an accompanying point of care diagnostic for detection of G 6 PD deficiency and can be deployed in districts with predominant vivax malaria with refractory malaria.

During the Covid-19 pandemic, countries including India mounted agile responses and made their regulatory and approval processes flexible. They adopted newer diagnostics, treatments and vaccines in the light of new evidence emerging via research. These efforts stemmed the pandemic significantly. For malaria elimination, we need to revive the same spirit.

^1^ Llanos-Cuentas, A., Lacerda, M. V., Rueangweerayut, R., Krudsood, S., Gupta, S. K., Kochar, S. K., Arthur, P., Chuenchom, N., Möhrle, J. J., Duparc, S., Ugwuegbulam, C., Kleim, J. P., Carter, N., Green, J. A., & Kellam, L. (2014). Tafenoquine plus chloroquine for the treatment and relapse prevention of Plasmodium vivax malaria (DETECTIVE): A multicentre, double-blind, randomised, phase 2b dose-selection study. The Lancet, 383(9922), 1049–1058. https://doi.org/10.1016/S0140-6736(13)62568-4

**Minor comments:**

12. The title – I would typically associate ‘tracking cases’ with individual patient data. However, the paper presents data on aggregate for district level. Could the title be changed to something that is more specific to the data presented for example (and *really* only an example) - ‘Tracking district level performance in the context of achieving zero indigenous case status by 2027’?

**Response:** As suggested, title has been modified now.

13. Page 4 -6: Long form of the RoR calculation and example of RoR calculation – I wonder whether these would be better placed in an annex

**Response:** As suggested, we adding these annexure as a web appendix.

14. Page 6 – where it reads “Where 'r' is the RoR in percentage (negative to indicate reduction) and 7 is the time component (from 2020 to 2027). Should the first brackets read ‘a negative number indicates reduction)? Otherwise reads like the question should be written as ‘-‘rather than ‘+’.

**Response:** As suggested, necessary modifications have been made.

15. Page 8 – First two lines of ‘results’ section should be in the methods section rather than results.

**Response:** As suggested, necessary modifications have been made.

16. Results section: The line ‘Therefore, the year of achieving zero cases status is hard to predict until they don't resume their negative growth (Figure 1).’ Reads a little strangely – is there any way to rephrase this? Also suggest spelling out ‘don’t’

**Response:** As suggested, necessary modifications have been made.

17. Page 11 – Sentence ‘The Comprehensive Case Management Programme, a joint venture in public-private partnership mode by ICMR-NIMR, Medicines for Malaria Venture (MMV) and the state government, identified poor surveillance and asymptomatic reservoirs as the major reasons for the persistence of the high malaria burden in the state’ seems strange to provide the detail on ‘public-private partnership by ICMR-NIMR and MMV’. Would read better if the sentence was shortened to ‘The Comprehensive Case Management Programme, a programme implemented by the state government, identified poor surveillance and asymptomatic reservoirs as the major reasons for the persistence of the high burden of malaria there (ref)’

**Response:** As suggested, necessary modifications have been made.

18. Page 11 – please spell out ‘ATSB’

**Response:** As suggested, full form has been added now

19. Page 11 – to whom are the several solutions proposed?

**Response:** All the solution if for National Programme that have been added in MS.

******************************

**Reviewer 2 comments in main file**

******************************

Reviewer #2: 1. Not all conclusions are supported by the data presented. Suggestions made in recommendation as to how to potentially make conclusions more specific to the results.
 Response: All suggested changes have been incorporated in the manuscript

3. Not all data are available publicly. The authors state that 'most' is available - it would be useful to identify what data is available publicly and where those could be found.

Response: The data availability statement has been changed accordingly.
