# Supplementary material for: Tracking district-level performance in the context of achieving zero indigenous case status by 2027
Source: PLOS Glob Public Health. 2023 Jan 6;3(1):e0001292. doi: 10.1371/journal.pgph.0001292 (PMC10021673; doi:10.1371/journal.pgph.0001292)
Supplement: Attachment — Submitted filename: Response to Reviewers_minor.docx [file pgph.0001292.s002.docx]

**Minor Comments**

Journal Requirements:

Please review your reference list to ensure that it is complete and correct. If you have cited papers that have been retracted, please include the rationale for doing so in the manuscript text, or remove these references and replace them with relevant current references. Any changes to the reference list should be mentioned in the rebuttal letter that accompanies your revised manuscript. If you need to cite a retracted article, indicate the article’s retracted status in the References list and also include a citation and full reference for the retraction notice.

**Response:** We confirm that we have not cited any retracted article, only those references have been cited which are relevant for this study.

Additional Editor Comments (if provided):

The responses to the comments are satisfactory; however, the authors need to speak to these issues in the manucript as caveats or limitations, and this hasn't consistently been done.

These need to be mentioned/ discussed in the manuscript, i.e. why the authors cannot conduct any validation or sensitivity analysis and the limitation of the simplified approach- not accounting for other factors etc, the limitations of excluding cases from the private sector and the potential bias of this in their outcomes of zero malaria cases. Or the comment on suggested interventions e.g the use of TQ as a solution for patient adherence. All these need to be expanded in the discussion and not only addressed as responses to reviewer comments.

**Response:** Issues regarding validation/sensitivity analysis, excluding cases from the private sector have been discussed in the 'Limitations' section now and points regarding TQ have been addressed in discussion section. All other important issues have already been discussed at appropriate places in the manuscript.

While revising your submission, please upload your figure files to the Preflight Analysis and Conversion Engine (PACE) digital diagnostic tool, https://pacev2.apexcovantage.com/. PACE helps ensure that figures meet PLOS requirements. To use PACE, you must first register as a user. Registration is free. Then, login and navigate to the UPLOAD tab, where you will find detailed instructions on how to use the tool. If you encounter any issues or have any questions when using PACE, please email PLOS at figures@plos.org. Please note that Supporting Information files do not need this step.

**Response:** All images were corrected by the tool suggested by you, we are very thankful for it.
